# Predictors of activity and participation across neurodegenerative conditions: a comparison of people with motor neurone disease, multiple sclerosis and Parkinson’s disease

**DOI:** 10.1186/s12883-018-1024-5

**Published:** 2018-02-17

**Authors:** David Morley, Sarah Dummett, Laura Kelly, Ray Fitzpatrick, Crispin Jenkinson

**Affiliations:** 1 Nuffield Department of Population Health, University of Oxford, Old Road Campus, Oxford, OX3 7LF UK; 2 Health Services Research Unit, Nuffield Department of Population Health, University of Oxford, Old Road Campus, Oxford, OX3 7LF UK

**Keywords:** Activity, Motor neurone disease, Multiple sclerosis, Neurodegenerative disease, Parkinson’s disease, Participation, Social isolation, Social engagement

## Abstract

**Background:**

Comparisons between neurological conditions have the potential to inform service providers by identifying particular areas of difficulty experienced by affected individuals. This study aimed to identify predictors of activity and participation in people with motor neurone disease (MND), people with multiple sclerosis (MS) and people with Parkinson’s Disease (PD).

**Methods:**

The Oxford Participation and Activities Questionnaire (Ox-PAQ) and Medical Outcomes Study 36-Item Short Form Survey (MOS SF-36) were administered by postal survey to 386 people with a confirmed diagnosis of MND, MS or PD. Data analyses focused on stepwise regression analyses in order to identify predictors of activity and participation in the three conditions assessed.

**Results:**

Three hundred and thirty four participants completed the survey, a response rate of 86.5%. Regression analyses identified multiple predictors of activity and participation dependent on Ox-PAQ domain and disease group, the most prominent being social and physical functioning as measured by the MOS SF-36.

**Conclusions:**

Results indicate that the physical and social consequences of neurological illness are of greatest relevance to people experiencing the conditions assessed. Whilst the largely inevitable physical implications of disease take hold, emphasis should be placed on the avoidance of social withdrawal and isolation, and the maintenance of social engagement should become a significant priority.

## Background

Comparisons between neurological conditions have the potential to enlighten service providers by identifying particular areas of difficulty experienced by affected individuals. This in turn can inform decision makers with important information on which to base, for example, allocation of resources. The focus of this study was a comparison of three progressive, neurodegenerative conditions; motor neurone disease (MND), multiple sclerosis (MS) and Parkinson’s disease (PD), where deterioration over time may lead to increased need for services.

Whilst MND, MS and PD sit under the broad umbrella of neurological disease, their clinical characteristics are markedly different. For example, MND is characterised by rapid deterioration of both upper and lower motor neurons, causing progressive muscle weakness and a precipitous pathway to severe disability. Although atypical forms of MND have been identified, most people with MND (PwMND) die of respiratory failure within 3 years [1]. By contrast, MS is a condition typified by recurrent relapses and remissions, although a small proportion of people with MS (PwMS) develop a chronic progressive form. Clinical symptoms can vary widely, including pain, fatigue, depression, and mobility and visual impairment [2–4]. PD is largely defined by the clinical features of tremor, bradykinesia and rigidity. People with Parkinson’s (PwP) can experience a range of other symptoms, both physical (e.g. falls, freezing of gait, hyperhidrosis) and cognitive (e.g. depression, hallucination, confusion) [5, 6].

Given the characteristics of the conditions outlined above, all three have the potential to have a significant impact on the well-being of people in a number of distinct ways. However, comparisons between PwMND, PwMS and PwP are rare. One exception to this is the prolific reporting of a data set from Australia that also includes a small sample of participants with Huntington’s disease. Data from this cohort, which includes caregivers, focuses on a range of pertinent factors including comparisons of quality of life (QoL), resilience, satisfaction with services, economic pressure, marital relationships and work and recreational changes [7–13].

To date, however, no study has adequately compared the impact of MND, MS and PD in the area of activity and participation. With an ageing population and advances in medicine that extend life expectancy in a range of chronic conditions, there is increasing emphasis on the need to keep people both active and participating in daily life [14]. Whilst McCabe et al. (2008) touch on this in relation to people with MND, MS and PD in their study of work and recreational changes [12], their study is qualitative in nature and thus the findings cannot be generalised. This study aims to make the first quantitative comparison of people with MND, MS and PD by identifying predictors of activity and participation in each condition and, in doing so, highlight implications for service providers.

## Methods

### Design and setting

This was a cross-sectional, postal survey that formed the basis of a previously reported validation study [15]. The Medical Sciences Inter Divisional Research Ethics Committee of the University of Oxford granted ethical approval for the validation work undertaken (reference MSD-IDREC-C1-2014-089).

### Participants

Recruitment of PwMND, PwMS and PwP was undertaken via their relevant support organisations in the United Kingdom; the Motor Neurone Disease Association, MS Society and Parkinson’s UK. The study was promoted through a range of media, including websites, social media, research bulletin boards, print and electronic publications, and email lists as a means of inviting individuals to contact the research team to express their interest in participating. Participants were required to be aged 18 years and above, competent in the use of English, living in the UK, have a diagnosis of MND, MS or PD and be able to complete the survey independently.

### Materials

A survey booklet containing three sections was administered by post:

- *Section 1*: demographic questions captured gender, age, age at diagnosis, marital status and ethnic origin.
- *Section 2:* The Oxford Participation and Activities Questionnaire (Ox-PAQ), a 23 item measure, comprising three domains; Routine Activities, Emotional Well-Being and Social Engagement. The recently validated Ox-PAQ is theoretically grounded in the World Health Organisation International Classification of Functioning, Disability and Health [16] and is specifically designed to assess participation and activity in people experiencing a range of health conditions. Items are answered on a five-point Likert scale, scores being transformed to a range of from 0 to 100 with higher scores indicating greater problems with activity and participation. Validation data indicates that the Ox-PAQ is both reliable and valid [15, 17].
- *Section 3*: Medical Outcomes Study 36-Item Short-Form Health Survey (MOS SF-36) [18, 19], a 36-item measure of health status comprising eight domains; Physical Functioning, Role Physical, Role Emotional, Social Functioning, Mental Health, Energy/Vitality, Pain and General Health Perception. Response options vary across items from dichotomous yes/no responses, up to a six-point Likert Scale. Raw scores for each health domain are transformed to have a range from 0 to 100 with higher scores indicating superior health status. The MOS SF-36 has been utilised in numerous research studies and has been shown to possess excellent psychometric properties [20].

### Procedure

The survey booklet and a consent form were posted to participants for completion and return. Reminders were sent to non-responders after 2 weeks via email or letter.

### Statistical analyses

Data were checked for normality of distribution and presence of outliers prior to statistical analysis. Stepwise regression analyses were conducted with the MOS SF-36 and demographic variables of age and disease duration in order to identify predictors of activity and participation across the three conditions. Data were analysed using SPSS Version 22 [21].

## Results

Three hundred and thirty four participants returned their completed questionnaire booklet, a response rate of 86.5%. The mean age was 60.06 years (standard deviation (SD) 12.10; range 24-88), the mean age at diagnosis was 52.82 years (SD 14.50; range 18-87) and the mean disease duration 7.31 years (SD 7.52; range 0–50). The sample comprised 162 males (48.5%) and 172 females (51.5%). Sample characteristics by disease group are detailed in Table 1.

**Table 1:** Sample characteristics by disease group

Condition	*N*=	Male: Female	Mean age (years)	Mean age at diagnosis (years)	Mean disease duration (years)
MND	97	65:32	62.96 *(8.93; 42-80)*	60.18 *(9.66; 35-78)*	2.78 *(4.11; 0-27)*
MS	100	21:79	49.12 *(11.26; 24-80)*	36.98 *(9.71; 18-63)*	11.94 *(9.32; 0-50)*
PD	137	76:61	66.12 *(8.83; 40-88)*	59.60 *(10.35; 30-87)*	6.93 *(5.57; 0-31)*
Total Sample	334	162:172	60.06 *(12.10; 24-88)*	52.82 *(14.50; 18-87)*	7.31 *(7.52; 0-50)*

(standard deviation; range)

### Predictors of activity and participation by disease group

Means and standard deviations for the eight MOS SF-36 domains that act as independent variables in the stepwise regression analyses are detailed in Table 2. Values for the additional variables of age and disease duration can be viewed in Table 1.

**Table 2:** Mean MOS SF-36 scores and standard deviations (in parentheses) by disease group

	Physical Functioning	Role Limitation Physical	Role Limitation Emotional	Energy/Fatigue	Emotional Well-Being	Social Functioning	Pain	General Health
MND	19.58 (28.33)	13.66 (26.76)	61.85 (43.30)	31.24 (22.55)	64.54 (20.40)	41.24 (31.15)	61.47 (27.89)	25.00 (17.78)
MS	37.85 (33.05)	18.43 (28.48)	57.33 (44.21)	34.10 (22.63)	64.00 (20.04)	53.62 (29.21)	62.75 (28.30)	38.06 (22.04)
PD	58.28 (27.51)	30.11 (37.27)	67.88 (40.71)	48.58 (22.14)	71.97 (16.98)	65.60 (28.35)	63.79 (26.34)	44.05 (21.10)

#### Predictors for PwMND

Analysis of Routine Activities identified two significant predictors explaining 78.8% of variance (R^2^ = .79, F (2,76) = 146.22, *p* < .001). The two predictors identified were Physical Functioning (β = −.71, p < .001), and Social Functioning (β = −.28, *p* < .001). Analysis of Emotional Well-being identified two significant predictors explaining 68.5% of the variance (R^2^ = .69, F (2,83) = 93.53, *p* < .001). The two predictors identified were MOS SF-36 Emotional Well-Being (β = −.76, p < .001), and Physical Functioning (β = −.21, *p* < .01). Analysis of Social Engagement identified two significant predictors explaining 51.4% of the variance (R^2^ = .53, F (2,80) = 44.35, *p* < .001). The two predictors identified were Social Functioning (β = −.58, *p* < .001), and Role Limitation Emotional (β = −.26, *p* < .01). Further regression statistics for PwMND can be viewed in Table 3.

**Table 3:** MOS SF-36 predictors of Ox-PAQ domains for people with motor neurone disease

Routine Activities	β	*R*	*R* ^2^	*R*^2^ Change	Adjusted *R*^2^
Physical Functioning	−.71	.86	.74	–	.74
Social Functioning	−.28	.89	.79	.05	.79
Emotional Well-Being	β	*R*	*R* ^2^	*R*^2^ Change	Adjusted *R*^2^
Emotional Well-Being	−.76	.81	.65	–	.65
Physical Functioning	−.21	.83	.69	.04	.68
Social Engagement	β	*R*	*R* ^2^	*R*^2^ Change	Adjusted *R*^2^
Social Functioning	−.58	.69	.47	–	.46
Role Limitation Emotional	−.26	.72	.53	.06	.51

#### Predictors for PwMS

Analysis of Routine Activities identified three significant predictors explaining 83.2% of variance (R^2^ = .84, *F* (3,87) = 149.15, *p* < .001). The three predictors identified were Physical Functioning (β = −.61 *p* < .001), Social Functioning (β = −.31, *p* < .001) and Pain (β = −.13, *p* < .05). Analysis of Emotional Well-being identified three significant predictors explaining 76.0% of the variance (R^2^ = .77, F (3,92) = 101.23, *p* < .001). The three predictors identified were MOS SF-36 Emotional Well-Being (β = −.68, p < .001), Pain (β = −.18, *p* < .01) and Energy /Fatigue (β = −.16, *p* < .05). Analysis of Social Engagement identified three significant predictors explaining 59.9% of the variance (*R*^2^ = .61, F (3,91) = 47.78, *p* < .001). The three predictors identified were Social Function (β = −.41, *p* < .001), Role Limitation Emotional (β = −.30, p < .001) and Physical Functioning. (β = −.28, *p* < .01). Further regression statistics for PwMS can be viewed in Table 4.

**Table 4:** MOS SF-36 predictors of Ox-PAQ domains for people with multiple sclerosis

Routine Activities	β	*R*	*R* ^2^	*R*^2^ Change	Adjusted *R*^2^
Physical Functioning	−.61	.86	.74	–	.74
Social Functioning	−.31	.91	.83	.09	.82
Pain	−.13	.92	.84	.01	.83
Emotional Well-Being	β	*R*	*R* ^2^	*R*^2^ Change	Adjusted *R*^2^
Emotional Well-Being	−.68	.84	.71	–	.70
Pain	−.18	.87	.75	.04	.75
Energy/Fatigue	−.16	.88	.77	.02	.76
Social Engagement	β	*R*	*R* ^2^	*R*^2^ Change	Adjusted *R*^2^
Social Functioning	−.41	.72	.51	–	.51
Role Limitation Emotional	−.30	.75	.56	.05	.55
Physical Functioning	−.28	.78	.61	.05	.60

#### Predictors for PwP

Analysis of Routine Activities identified three significant predictors explaining 75.8% of variance (*R*^2^ = .76, *F* (3,118) = 127.54, *p* < .001). The three predictors identified were Physical Functioning (β = −.48, *p* < .001), Social Functioning (β = −.34, p < .001) and Emotional Well-Being (β = −.22, *p* < .001). Analysis of Emotional Well-being identified three significant predictors explaining 69.5% of the variance (R^2^ = .70, F (3,121) = 95.35, *p* < .001). The three predictors identified were MOS SF-36 Emotional Well-Being (β = −.65, *p* < .001), Role Limitation Physical (β = −.18, *p* < .01) and Physical Functioning (β = −.16, *p* < .01). Analysis of Social Engagement identified three significant predictors explaining 58.4% of the variance (*R*^2^ = .59, F (3,121) = 58.96, *p* < .001). The three predictors identified were Social Functioning (β = −.39, *p* < .001), Emotional Well-Being (β = −.29, *p* < .001) and Physical Functioning (β = −.24, *p* < .01). Further regression statistics for PwP can be viewed in Table 5.

**Table 5:** MOS SF-36 predictors of Ox-PAQ domains for people with Parkinson’s disease

Routine Activities	β	*R*	*R* ^2^	R^2^ Change	Adjusted *R*^2^
Physical Functioning	−.48	.78	.61	–	.60
Social Functioning	−.34	.86	.73	.12	.73
Emotional Well-Being	−.22	.87	.76	.03	.76
Emotional Well-Being	β	*R*	R^2^	*R*^2^ Change	Adjusted *R*^2^
Emotional Well-Being	−.65	.80	.63	–	.63
Role Limitation Physical	−.18	.83	.69	.06	.68
Physical Functioning	−.16	.84	.70	.01	.69
Social Engagement	β	*R*	*R* ^2^	*R*^2^ Change	Adjusted *R*^2^
Social Functioning	−.39	.70	.49	–	.48
Emotional Well-Being	−.29	.75	.56	.07	.55
Physical Functioning	−.24	.77	.59	.03	.58

## Discussion

This study has aimed to identify predictors of activity and participation via the MOS SF-36 as a means of providing insight into how each condition might be managed in order to maintain activity and participation in people experiencing the three conditions assessed.

For PwMND results suggest that maintaining physical and social functioning, as measured by the SF-36, is key to well-being in terms of activity and participation as measured by the Ox-PAQ. Maintaining physical functioning is clearly challenging with the rapid and severe decline associated with MND. Social functioning, however, has the potential to be addressed through the avoidance of social isolation that many PwMND experience [22], and it has been suggested that interventions should be developed to facilitate this [23].

For PwMS results similarly suggest that physical and social functioning are important predictors of activity and participation, whilst also highlighting the added significance of pain and fatigue. Physical and social functioning have previously been recognised as significant factors in the well-being of PwMS [24, 25]. Pain and fatigue have also been identified as critical to the well-being of PwMS [26–28] in what is a complex picture in the management of the condition, particularly given that 85% experience the relapsing, remitting form of the condition [29].

For PwP results again highlight physical and social functioning as seen in previous research [30], whilst also drawing attention to the added significance of emotional well-being. Depression in PwP is well documented across the literature [31, 32] and strategies to alleviate its deleterious effects should be prominent in the management of PD.

A number of limitations from this study are acknowledged. Firstly, this is a cross-sectional study, thereby only providing an assessment at one point in time and, although adequate for the analyses conducted, sample sizes recruited are relatively small. Further research in this area should aim to incorporate longitudinal methodology and recruit larger samples in order to gain a greater understanding of the impact of neurodegenerative disease on activity and participation over time. Larger samples would also allow for comparisons by disease stage and clinical condition, which may make an informative line of research. Additionally, it is recognised that neurodegenerative diseases demonstrate significant heterogeneity in clinical characteristics, especially so in MND, and that the proportion of different subtypes in each disease group may have affected the results. It is also acknowledged that data has been collected by way of self-report, which can lead to potential biases in results. Finally, participants from each of the three conditions assessed were self-selecting, and therefore may not be entirely representative of their particular disease group.

## Conclusions

Studies such as that reported here are relatively rare and may be a useful indicator to services as to which areas of peoples’ lives might be best targeted to maintain or enhance their ability to engage and participate in meaningful activities of daily life. Overwhelmingly, the results presented suggest that it is the physical and social consequences of neurodegenerative disease that are of greatest relevance. That the latter is often overlooked should be of greatest concern. Whilst the largely inevitable physical consequences of disease take hold, social withdrawal and isolation should be viewed as neither inevitable nor intractable. Ameliorating the stigma associated with neurological conditions may go some way towards addressing this. For example, stigmatisation in PwP is well documented [33–37] and acts as a barrier to maintaining social engagement. Consequently, greater openness and understanding of the challenges faced by people experiencing neurological illness should be promoted, alongside the development of appropriate interventions, in order to aid their social engagement.

## Abbreviations

MND: Motor neurone disease
MOS SF-36: Medical Outcomes Study 36-Item Short Form Survey
MS: Multiple sclerosis
Ox-PAQ: Oxford Participation and Activities Questionnaire
PD: Parkinson’s disease
PwMND: People with motor neurone disease
PwMS: People with multiple sclerosis
PwP: People with Parkinson’s
QoL: Quality of life
SD: Standard deviation
